# Incontinence in Individuals with Rett Syndrome: A Comparative Study

**DOI:** 10.1007/s10882-012-9271-7

**Published:** 2012-02-23

**Authors:** Sanne Giesbers, Robert Didden, Maartje Radstaake, Hubert Korzilius, Alexander von Gontard, Russell Lang, Eric Smeets, Leopold M. G. Curfs

**Affiliations:** 1 Behavioural Science Institute, Radboud University Nijmegen, PO Box 9104, Nijmegen, 6500 HE The Netherlands; 2 Institute for Management Research, Radboud University Nijmegen, Nijmegen, the Netherlands; 3 Department of Child and Adolescent Psychiatry, Saarland University Hospital, Homburg, Germany; 4 Clinic for Autism Research Evaluation and Support, Texas State University, San Marcos, TX USA; 5 Department of Clinical Genetics, University of Maastricht, Maastricht, the Netherlands

**Keywords:** Rett syndrome, Incontinence, Comparative study

## Abstract

Frequency and type of incontinence and its association with other variables were assessed in females with Rett Syndrome (RS) (*n* = 63), using an adapted Dutch version of the ‘Parental Questionnaire: Enuresis/Urinary Incontinence’ (Beetz et al. 1994). Also, incontinence in RS was compared to a control group consisting of females with non-specific (mixed) intellectual disability (*n* = 26). Urinary incontinence (UI) (i.e., daytime incontinence and nocturnal enuresis) and faecal incontinence (FI) were found to be common problems among females with RS that occur in a high frequency of days/nights. UI and FI were mostly primary in nature and occur independent of participants’ age and level of adaptive functioning. Solid stool, lower urinary tract symptoms and urinary tract infections (UTI’s) were also common problems in females with RS. No differences in incontinence between RS and the control group were found, except for solid stool that was more common in RS than in the control group. It is concluded that incontinence is not part of the behavioural phenotype of RS, but that there is an increased risk for solid stool in females with RS.

Rett Syndrome (RS) is a progressive neurodevelopmental disorder found primarily in females. RS occurs worldwide with an incidence of 1.09 per 10.000 females by the age of 12 years (Laurvick et al. 2006), and is considered to be the second most frequent cause of severe intellectual disability (ID) in females. MECP2 (methyl-cpG-binding protein 2) gene mutations on the X chromosome can be identified in up to 90% of individuals with RS (Smeets et al. 2003). RS is clinically defined by criteria revised in 2010 (Neul et al. 2010), and is characterized by a progressive decline in psychomotor and adaptive functioning starting early in life after a period of seemingly normal development. Clinical features that emerge are severe ID, stereotyped hand movements, social withdrawal, communication impairment, impaired or failing locomotion, seizures, breathing abnormalities, and scoliosis.

In diagnosis, typical and atypical RS are differentiated. Typical RS is divided into four stages (Hagberg 2002; Lavas et al. 2006). The stagnation stage (stage 1) is characterized by a general development stagnation that starts when the child is between 5 and 18 months. The onset of the regression stage (stage 2) is usually between 1 and 3-4 years. In this stage there is loss of acquired skills and acquired communication, such as hand function, the ability to speak, contact, social interaction, and play, though eye contact for communicative purposes is preserved (see e.g., Didden et al. 2010; Sigafoos et al. 2011). Stereotypical hand movements (e.g., wringing, clapping, mouthing), breathing abnormalities, and behavioral disturbances (e.g., screaming, laughing and crying attacks during the night) emerge. The stationary stage (stage 3) generally begins between 2 and 10 years of age and includes apraxia, motor problems, scoliosis and seizures. There may also be an improvement in behavior and social and communicative skills. Some females remain in this stage for the rest of their lives. The motor deterioration stage (stage 4) starts after the age of 10 and is characterized by severe physical disability, reduced mobility, and a decrease of stereotyped hand movements.

Although incontinence is found to be common among individuals with severe ID, studies on incontinence and associated variables in individuals with RS have not been published yet. According to the International Children’s Continence Society (ICCS) standardization document (Nevéus et al. 2006), Urinary Incontinence (UI) is the uncontrollable leakage of urine, which can be continuous or intermittent. Intermittent incontinence can be differentiated into Nocturnal Enuresis (NE) and Daytime Incontinence (DI). NE is incontinence in discrete amounts during sleep and can be differentiated into primary (never been dry) or secondary (relapse after a dry period of at least 6 months), as well as mono- (without) and nonmonosymptomatic (with lower urinary tract symptoms). DI is incontinence in discrete amounts during the day. Dual diagnoses are given to children with combined day- and night-time UI. With regard to Faecal Incontinence (FI), the Rome III classification differentiates between functional constipation (with or without FI) and non-retentive FI (incontinence without constipation) (Rasquin et al. 2006).

As stated, incontinence is common in individuals with severe ID. Rates of incontinence are higher with level of ID becoming more severe (Bruschini et al. 2003; Saloviita 2002; Yang et al. 2010). For instance, in the study of Saloviita (2002) it was noted that the percentages of NE among individuals with moderate, severe, and profound ID were 6%, 16%, and 45%, respectively. Also, level of physical impairment is positively associated with rates of incontinence (Quander et al. 2006; Van Laecke et al. 2010).

Incontinence may have physical and social consequences that adversely affect the individual’s quality of life. For instance, perineal dermatitis, an inflammatory condition of the skin in the perineal area, is a common problem for individuals with urinary and faecal incontinence. Perineal dermatitis not only can cause pain and itching, it also increases the susceptibility to urinary tract infection (UTI) (Driver 2007). Among the social consequences are stigmatization, difficulties in social relationships, and reduced opportunities for integration (e.g., less access to community based activities and school) (Grey and McClean 2007). Incontinence not only adversely affects the quality of life of the individuals themselves, it may also place a significant physical and psychological burden on parents and other caregivers (Gotoh et al. 2009; Landefeld et al. 2008). Finally, economic costs for incontinence are substantial (Borrie and Davidson 1992; Miner 2004; Landefeld et al. 2008). These costs include, for instance, medical health care, nursing time, laundry, and incontinence supplies.

There has been increasing recognition that genetic disorders are associated with specific behaviors, termed ‘behavioral phenotype’. A behavioral phenotype is best described as the heightened probability or likelihood that individuals with a specific genetic syndrome will exhibit particular behaviors compared to others without the syndrome (Dykens 1995; Hodapp and Dykens 2005). However, incontinence has only rarely been examined in individuals whose ID is associated with a genetic disorder. For instance, Backes et al. (2000) showed that 27% of individuals with Fragile X syndrome (at a mean age of 8.5 years) had UI, while 20% had FI. For individuals with tuberous sclerosis, the rates for UI and FI (at a mean age of 9.5 years) were 13% and 6%. Recently, Von Gontard et al. (2010) found that 13.6% of individuals with Prader-Willi syndrome (at a mean age of 15.1 years) had NE, 3.8% had additional DI, and 3.3% had FI.

Although an increasing number of studies on the behavioral phenotype of RS have been published, no studies with a main focus on incontinence in RS have been conducted yet. In one study it was noted that 67.6% of individuals with RS was incontinent, while 28.2% was partially toilet trained (Cass et al. 2003), but in this study only an overall prevalence rate was determined and a control group was not included. Furthermore, diagnostic criteria and reviews of (behavioral) features of RS largely fail to include information on incontinence. In the exceptional case of a reference to incontinence in those reviews, constipation is mentioned, but these observations were merely clinical in nature (Hagberg 2002). Systematic studies on incontinence in RS—to examine whether RS is associated with specific aspects of incontinence—are crucial, given the aforementioned adverse consequences.

Therefore, the purpose of the present study was to provide data on incontinence and associated variables in individuals with RS. More specifically, the aim of the present study was to (a) explore frequency and type of incontinence in a sample of individuals with RS (*n* = 63), (b) explore associations between incontinence and other variables (e.g., type and cause of RS, epilepsy, living setting), and (c) compare RS to a matched control group. Incontinence is considered to be part of the behavioral phenotype of RS if it is found to be present in a greater proportion of girls with RS as compared to a control group of individuals with mixed ID matched for gender, age, and level of adaptive functioning (Hodapp and Dykens 2001).

## Method

### Participants

#### Rett Syndrome

Participants were 63 females with a mean age of 19.34 years (*SD* = 11.39; range 5.08–47.17 years) and a mean level of adaptive functioning of 9 months (*SD* = 8; range: 2–38 months). Of the participants, 45 (80.4%) had classic or typical RS and 11 (19.6%) had variant or atypical RS. MECP2 mutations were confirmed in 49 (79.0%) cases. 42 (66.7%) females had epilepsy of which 16 (38.1%) were seizure-free as a result of anticonvulsive medication. Breathing abnormalities were present in 46 (73.0%) participants of whom 24 (52.2%) had hyperventilation, 31 (72.1%) showed shallow breathing, 40 (87.0%) had breath holding spills, and 22 (48.9%) showed forced expulsion of air. Thirty-eight (60.3%) females were wheelchair bound and scoliosis was present in 48 (76.2%) cases. Forty (63.5%) participants lived at home.

#### Control Group

Participants were 26 females with a mean age of 21.64 years (*SD* = 11.54; range: 5.25–47.33) and a mean level of adaptive functioning of 8 months (*SD* = 7; range: 2–23 months). Eleven (45.8%) participants had a known cause for their disability, for instance oxygen deprivation at birth (hypoxia). Of the participants, 8 (33.3%) lived at home. Eighteen (69.2%) females had epilepsy of which 6 (33.3%) were seizure-free as a result of anticonvulsive medication and 17 (65.4%) females were wheelchair bound.

### Materials

#### Incontinence Questionnaire

The incontinence questionnaire was adapted from the ‘Parental Questionnaire: Enuresis/Urinary Incontinence’ (Beetz et al. 1994) and translated into Dutch. The questionnaire consists of 43 items referring to day- and nighttime wetting (e.g., ‘How many days a week does your child/client wet his/her clothes or diaper’), toileting (e.g., Does your child/client go to the toilet him/herself if he/she needs to or does he/she express that he/she wants to go to the toilet), observable voiding behaviors and reactions (e.g., ‘When your child/client needs to void, does he/she have to rush to the toilet immediately), urinary tract function (e.g., ‘Does your child/client ever had a UTI’) stool habits (e.g., ‘Does your child/client have daily bowel movements’) and behavioral symptoms (e.g., ‘Does your child/client wet more often in stressful times’) which have to be filled out by primary care-givers in a yes/no format. Additional data (such as voiding and defecation frequency) are noted numerically. In some questions, parents are asked for clarification. The questionnaire is suitable for children who are at least 5 years of age. It has been used in a study on children with Spinal Muscular Atrophy (SMA) (Von Gontard et al. 2001), and, recently, in a study on individuals with PWS (Von Gontard et al. 2010).

#### Vineland Screener 0–6 years

Level of adaptive functioning was measured through the Dutch version of the Vineland Screener 0–6 years (Scholte et al. 2008). The Vineland Screener 0–6 years is a validated questionnaire that measures adaptive behavior in individuals with a developmental level to 6 years across four domains (i.e., communication, daily living skills, socialization, and motor skills). The questionnaire consists of 72 items which have to be filled out by primary care-givers. Care-givers have to indicate on a 3-point scale from 0 (no, never) to 2 (yes, usually) whether the skills described in the items are performed independently by the individual (e.g., ‘Does he/she open locks with a key’). Vineland Screener scores could be converted into an adaptive developmental age for both the total scale and the four domains separately. Psychometric properties of the screener have been assessed in individuals between 1 and 18 years who visit a day care center for individuals with ID in the Netherlands. Results indicate good internal consistency (i.e., Cronbach alpha for the four domains is .86 or higher) and construct validity.

### Procedure

Questionnaires (see Materials) were sent to parents who were members of the Dutch Rett Syndrome Association and who had a child with RS. In an accompanying letter, parents were asked to fill out and return the questionnaires. Non-respondents were sent a reminder after 5 weeks. Out of 169 questionnaires that were sent to parents, 73 were returned (response rate: 43%). Ten individuals were excluded due to incomplete data or age constraints. Solely individuals from 5 to 55 years were included in this study, since incontinence is diagnosed from 5 years of age (Nevéus et al. 2006). After the age of 55 other factors may account for incontinence, for instance dementia (Moss and Patel 1997).

Similarly to earlier studies on the behavioral phenotype of RS (Mount et al. 2002; Mount et al. 2003), a control group of individuals with mixed severe to profound ID (IQ < 35, level of adaptive functioning < 48 months) was recruited. These participants were recruited through three facilities for individuals with ID in the eastern and southern part of the Netherlands. Questionnaires were sent to parents or staff members of 142 individuals with mixed severe to profound ID. In an accompanying letter, parents or staff-members (depending on the living situation of the individual) were asked to fill out and return the questionnaires. Out of 142 questionnaires, 96 were returned (response rate: 68%). To match for gender, only females were included in this study (*n* = 41). One female was excluded due to age constraints (i.e. age > 55) and three females were excluded due to a level of adaptive functioning higher than 48 months.

Mean level of adaptive functioning of females with RS turned out to be significantly lower than mean level of adaptive functioning of the control group. To ensure that the two groups did not differ with regard to level of adaptive functioning, solely females with profound ID (IQ < 20, level of adaptive functioning < 24 months) were included in the between-group analyses. As a result, two females with RS and 11 females from the control group were excluded. Furthermore, due to missing data, level of adaptive functioning was not known for 10 individuals with RS and these females were also not included in the between-group analyses. Eventually, RS (*n* = 51) and the control group (*n* = 26) did not differ with regard to level of adaptive functioning (*t* (75) = -.304, *p* = .76). There were also no significant differences between the groups with regard to age (*t* (75) = -.1.171, *p* = .25), motor abilities (*t* (75) = -.801, *p* = .43), epilepsy (*χ²* = 0.120, *p* = .73), and being wheelchair bound (*χ²* = 0.052, *p* = .82).

### Statistical Analyses

Chi-squared tests were performed to test associations between incontinence and other variables within RS. Where associations could not be tested with Chi-squared tests, because of low cell frequencies, a two-tailed Fisher’s exact test was conducted. This test only produces a significance level and no formal test statistic. To test differences on ratio level data (i.e., age and level of adaptive functioning) independent sample *t*-tests were performed. To test differences in percentages of incontinence and related variables between RS and the control group, Chi-squared tests or, in case of low cell frequency, Fisher’s exact tests were conducted. To assess differences in the frequency of incontinence, independent samples *t*-tests were conducted. Missing values occurred in our data. In the case of only a few missing values, pairwise deletion was used, participants who had many missing values were excluded from the analyses (see Procedure).

## Results

### Descriptive Statistics

#### Rett Syndrome

Number, percentage, and frequency of various types of incontinence are shown in Table 1. DI and NE were present among almost all females with RS and the mean number of days/nights with incontinence was high. FI is somewhat less common, but nevertheless present in the majority of cases.

**Table 1:** Number, percentages and frequency of incontinence in Rett Syndrome (*n* = 63) and control group (*n* = 26)

		*n* (%)	Mean number of days, nights, times/week (*SD*)
DI	RS	61 (96.8)	6.9 days (0.8)
	Control	25 (96.2)	7.0 days (0.0)
NE	RS	60 (98.4)	7.0 nights (0.3)
	Control	25 (96.2)	6.9 nights (0.6)
FI–day	RS	44 (72.1)	5.9 times (4.2)
	Control	23 (88.5)	7.4 times (6.4)
FI–night	RS	35 (57.4)	1.4 times (1.2)
	Control	15 (57.7)	2.1 times (1.4)

*DI* Daytime incontinence, *NE* Nocturnal enuresis, *FI* Faecal incontinence, *RS* Rett syndrome

Out of 61 females with DI, five (8.2%) females previously had a dry period for a mean of eight years (*SD* = 11.46). Twenty-four (40.0%) females with DI also voided on the toilet with a mean of 2.5 times a day (*SD =* 1.64) and 15 (27.8%) females were reported to indicate that they have wetted by pulling or pointing to their pants/diaper or by making noises.

No females with NE have had a dry period before. Thirty-eight (80.9%) females were reported to wet large amounts during the night and three (5.5%) were reported to wake up after wetting. Eighteen (32.7%) females appeared to be deep sleepers. Prevalence of lower urinary tract symptoms are shown in Table 2 for females with NE who voided on the toilet, because these symptoms cannot be observed if the person solely wets the diaper.

**Table 2:** Lower urinary tract symptoms in females RS and NE who void on the toilet (*n* = 25)

	*n* (%)
Daytime Incontinence	24 (96.0)
Hesitancy	5 (23.8)
Straining	1 (4.0)
Weak Stream	5 (20.0)
Intermittency	8 (33.3)
Holding maneuvers	0 (0.0)
Post-micturition dribble	5 (21.7)

*NE* Nocturnal enuresis, *RS* Rett syndrome

Three (4.8%) females had been diagnosed with a medical condition in the urinary system (i.e., impaired bladder emptying, high calcium levels in the urine and kidney stones, and urethral stricture) for which they received treatment.

Thirty (47.6%) females have ever had a UTI (15 (57.7%) females with fever) with a mean frequency of 3.36 times (*SD =* 2.40). Twenty-eight (96.6%) females received treatment for their UTI in the form of antibiotics. Three of these females received antibiotic prophylaxes due to high frequent UTI’s and one female received additional bladder flushes.

Thirty-six (58.1%) females had daily bowel movements. Solid stool was present in 40 (64.5%) females and 48 (76.2%) females received medication to stimulate bowel movements. Out of 47 (77.0%) individuals with (daytime, nighttime or combined) FI, five (10.6%) previously had complete bowel control for a mean of 2.67 years (*SD =* 0.58).

Table 3 depicts number of individuals with RS who were incontinent in terms of behavior, impact of incontinence, efforts to stimulate continence, and reasons parents/caregivers give for the incontinence.

**Table 3:** Symptoms in females with RS with UI and/or FI (*n* = 62)

		*n* (%)
Behavior	Restless, on the go, easily distracted	27 (46.6)
	Anxious—general	36 (59.0)
	Anxious—toilet	3 (5.3)
Impact incontinence	Parent Distressed	6 (10.2)
	No access to activities	1 (1.7)
Continence stimulation	Efforts ever made	40 (65.6)
	Current efforts	21 (34.4)
Incontinence reasons	Intellectual disability	53 (89.9)
	No free access to toilet	9 (14.8)
	Medical reasons	4 (6.6)
	Syndrome	3 (4.9)
	Communication impairment	4 (6.6)

*UI* Urinary incontinence, *FI* Faecal incontinence, *RS* Rett syndrome

#### Control Group

Number, percentage, and frequency of various types of incontinence are shown in Table 1. Twelve (48.0%) females have ever had a UTI (8 (72.7%) females with fever) with of mean frequency of 3.29 times (*SD =* 1.80). Thirteen (54.2%) females had daily bowel movements and 19 (73.1%) females received medication to stimulate bowel movements. Solid stool was present in 8 (30.8%) females.

### Within-Group Analyses

Chi-squared or, in case of low cell frequency, Fisher’s exact tests were performed to test associations between incontinence and related variables (i.e., DI, NE, FI, UTI, daily stool, and solid stool) and participant characteristics (i.e., type and cause of RS, epilepsy, breathing abnormalities, wheelchair bound, scoliosis, and living setting) (Table 4). First, it was found that UTI’s were more common among females with epilepsy than among females without epilepsy (χ²(1) = 7.76, *p* = .01). Furthermore, compared to females without scoliosis, UTI’s were more common among females with scoliosis (χ²(1) = 4.67, *p* = .03) and daily stool was less common among these females (χ²(1) = 5.36, *p =* .02). Daily stool was also less common among females who did not live at home, compared to females who did live at home (χ²(1) = 8.14, *p* = .004). Lastly, FI during the night was more common among females who were ambulant than among females who were wheelchair bound (χ²(1) = 5.03, *p* = .03).

**Table 4:** Incontinence and participant characteristics, χ², and p-values

Variable	Participant Characteristics						
	Type of RS	Cause of RS	Epilepsy	Breathing Abnormalities	Wheelchair bound	Scoliosis	Living setting
DI	.36 (#)	.38 (#)	.53 (#)	1.00 (#)	.15 (#)	.05 (#)	.53 (#)
NE	1.00 (#)	.18 (#)	.68 (#)	1.00 (#)	.39 (#)	.23 (#)	1.00 (#)
FI-day	.71 (#)	.49 (#)	.07 (3.19)	.52 (#)	.68 (0.16)	.51 (#)	.61 (0.27)
FI-night	.20 (1.67)	.37 (#)	.32 (0.98)	.10 (2.75)	.03 (5.03)*	.97 (0.002)	.20 (1.64)
UTI	.74 (0.11)	.17 (#)	.01 (7.76)*	.61 (0.26)	.22 (0.64)	.03 (4.67)*	.11 (2.55)
Daily stool	.31 (#)	.11 (#)	.78 (0.25)	.94 (0.01)	.19 (1.70)	.02 (5.36)*	.004(8.14)*
Solid stool	1.00 (#)	.38 (#)	.34 (0.92)	.42 (0.64)	.18 (1.83)	.91 (0.01)	.51 (0.44)

# Fisher's exact test, * p < .05, *DI* Daytime incontinence, *NE* Nocturnal enuresis, *FI* Faecal incontinence, *UTI* Urinary tract infection, *RS* Rett syndrome

To test associations between incontinence and age and level of adaptive functioning, independent samples *t*-tests were conducted. (Due to lack of variance in DI and NE (i.e., almost all females with RS had DI and NE), it was not possible to perform *t*-tests for these variables.) No statistically significant differences were found in age and level of adaptive functioning between females with FI (day and night) and females without FI (day and night).

### Between-Group Analyses

Chi-squared tests or Fisher’s exact tests were conducted to assess differences between RS and the control group in percentages of incontinence and related variables (i.e., DI, NE, FI, UTI, daily stool, and solid stool). Results are shown in Table 5. There were no significant differences between groups, except for solid stool, which was more common in females with RS than in females of the control group (χ² (1) = 7.97, *p* = .01).

**Table 5:** Percentages for each variable by group, χ², and p-values

Variable	Group	*n* (%)	χ²	*p*
DI#	RS	51 (100)		.34
	Controls	25 (96.2)		
NE#	RS	50 (100)		.34
	Controls	25 (96.2)		
FI–day	RS	38 (76.0)	1.68	.20
	Controls	23 (88.5)		
FI–night	RS	30 (58.8)	0.001	.98
	Controls	15 (57.7)		
UTI	RS	23 (45.1)	0.06	.81
	Controls	12 (48.0)		
Daily stool	RS	29 (58.0)	0.10	.76
	Controls	13 (54.2)		
Solid stool	RS	33 (64.7)	7.97	*.*01*
	Controls	8 (30.8)		
Medication for stool	RS	38 (74.5)	0.02	.89
	Controls	19 (73.1)		

# Fisher's exact test, * *p* < .05, *DI* Daytime incontinence,

*NE* Nocturnal enuresis, *FI* Faecal incontinence, *UTI* Urinary tract infection, *RS* Rett syndrome

Independent samples *t*-tests were performed to test differences between RS and the control group in the frequency of DI, NE, FI, and UTI (Table 6). No statistically significant differences in frequency of these variables were found between groups, although two variables (i.e. NE and FI-night) approached significance level.

**Table 6:** Mean number of days/nights/times, t, and p-values

	Group	*n*	*M* (*SD*)	*df*	*t*	*p*
DI	RS	50	6.94 (0.4)	73	0.71	.48
	Controls	25	7.00 (0.0)			
NE	RS	50	6.96 (0.3)	73	0.79	.08
	Controls	25	6.88 (0.6)			
FI–day	RS	36	6.53 (4.3)	55	0.64	.55
	Controls	21	6.41 (7.4)			
FI–night	RS	22	1.22 (1.2)	32	1.93	.06
	Controls	12	2.07 (1.4)			
UTI	RS	18	3.28 (2.6)	23	0.01	.99
	Controls	7	3.29 (1.8)			

*DI* Daytime incontinence, *NE* Nocturnal enuresis, *FI* Faecal incontinence, *UTI* Urinary tract infection, *RS* Rett syndrome

## Discussion

This study is the first to investigate incontinence in individuals with RS and in which outcomes were compared to those of a control group. The results show that both UI (i.e., DI and NE) and FI are common problems among females with RS. Nearly all females had DI and NE. FI was somewhat less common, but still present in the majority of cases (i.e., 72% during daytime, 57% at night).

Despite the progressive nature of the syndrome, 8% of females with DI, none of females with NE, and 11% of females with FI had been dry before and relapsed, suggesting that incontinence has its onset in the earliest stages of the syndrome.

As 96% of females with NE had DI, most females with NE would be classified as non-monosymptomatic (Nevéus et al. 2006). Other lower urinary tract symptoms that were present among these females were hesitancy, weak stream, intermittency, and post-micturition dribble. It must be noted that these symptoms could only be observed in females who voided on the toilet, limiting the generalizability of these outcomes. Since dysfunctional voiding seems to be a problem in these females, urodynamic assessment of females with RS would be required (Nevéus et al. 2006). Also, dysfunctional voiding can be treated effectively (Chase et al. 2010).

Although not deviant from the incidence of UTI’s in females of the general (nondisabled) population (Foxman 2003), it is noteworthy that nearly half of females with RS have ever had a UTI. Incontinence (and lower urinary tract function) are a risk factor for UTI’s. UTI’s in turn, can be a cause of UI (Von Gontard and Neveus 2006). Since UTI’s can cause major discomfort and, if untreated, can develop into serious kidney infections that can permanently scar or damage the kidneys (Sweet and Gibbs 2009), it is important that they are detected and treated early. Due to their severe ID and communication impairment, females with RS may not be able to adequately communicate their discomfort. Caregivers should therefore be aware of this problem. In more than half of cases, the UTI was accompanied with fever, an indication that the infection has reached the kidneys (Sweet and Gibbs 2009).

Furthermore, solid stool was common among females with RS (i.e., 65%), a finding that indicates constipation. Possible contributing factors to the development of constipation is physical incapacitation (e.g., not being able to walk and lying position), as was typical in children with spinal muscular atrophy (Von Gontard et al. 2001). High prevalence of constipation would be in line with Hagberg’s (2002) review of features of RS. No females defecate two times a week or less, though stool retention is possible even with frequent bowel movements. Therefore, other symptoms than infrequent defecations are necessary to diagnose constipation adequately (Rasquin et al. 2006). Stool retention and constipation are also well known risk factors for the development of NE, DI, and FI (Von Gontard and Neveus 2006). Therefore, desimpaction and maintenance treatment with oral laxatives (especially polyethylene glycol) is recommended (Von Gontard 2010).

In contrast to parents of typically developing children (Chang et al. 2002; De Bruyne et al. 2009), most parents were not distressed by the incontinence despite its high frequency. It is suggested that in parents’ experience incontinence does not have major implications for the child in addition to their severe intellectual and physical disability. For instance, contrary to reports of parents of typically developing children, parents reported that incontinence did not influence their child’s access to activities (Morison 2000). Most parents attribute the incontinence to their child’s profound ID. This suggests that parents believe that incontinence ‘belongs’ to ID. However, in one-third of females, efforts to stimulate continence are still made. Moreover, despite their incontinence, 40% of females voided on the toilet daily, indicating that parents continue to provide toilet moments to their child.

66% of parents reported that efforts have been made to stimulate continence in their child. In most cases these efforts included scheduled toilet times but which remained ineffective. Many individuals with ID are not offered professional assessment and treatment of incontinence. Simple interventions such as increasing oral fluids or anticholinergic medication can reduce incontinence dramatically (Van Laecke et al. 2009). Furthermore, none of the parents reported that their child has received systematic toilet training. Toilet training has been shown effective in individuals with severe ID and in those with a genetic disorder (see e.g., Azrin and Foxx 1971; Averink et al. 2005; Didden et al. 2001). However, until now, no studies on the effectiveness of systematic toilet training in females with RS have been published.

Within-group analyses show that UTI’s were more common among females with epilepsy than among females without epilepsy. Furthermore, compared to females without scoliosis, UTI’s were more common among females with scoliosis, and daily stool was less common among these females. Daily stool was also less common among females who did not live at home, compared to females who did live at home. It may be suggested that at home, consumed food and toilet times coincide with individual needs in a better extent compared to, for instance, residential facilities. Lastly, FI during the night was more common among females who were ambulant than among females who were wheelchair bound.

When compared to a control group of females with non-specific (mixed) ID, UI (i.e., DI and NE) and FI, UTI’s, daily stool, and the use of medication to stimulate bowel movements did not differ in females with RS. However, solid stool was more common among females with RS than among females of the control group. Since no differences in incontinence between RS and individuals with non-specific ID have been found, it may be concluded that RS is not associated with an increased risk of incontinence, but that there is an increased risk of solid stool in females with RS.

Results of the present study must be interpreted in the context of the study’s methodological shortcomings. The first shortcoming is the reliance on parental report. Parental reports are subjective and a more objective method such as clinical and urodynamic assessment may have revealed specific functional disturbances (Nevéus et al. 2006). The second shortcoming concerns the relatively low response rate (43%) of the RS group (compared to the control group and a study using the same questionnaire (Von Gontard et al. 2010)), which may limit the generalizability of the outcomes. Non-respondents were not assessed and may not be comparable to participants in the study. For instance, parents of females who are incontinent may have valued the study more and were therefore more likely to participate than parents of females who did not face any of these problems. This may have affected the outcomes of the study. A third shortcoming is that sample size of the control group was smaller than sample size of RS. The small sample size of the control group might have contributed to the fact that significant differences between the two groups in incontinence were not found in this study. The last shortcoming relates to the use of the incontinence questionnaire. Although it has been used in earlier studies, psychometric properties of the questionnaire are unknown. In the future, psychometric research (i.e., reliability and validity research) on the incontinence questionnaire should be conducted.

From the current study it may be concluded that UI (i.e., DI and NE) and FI incontinence are common problems among females with RS and occur in a high frequency of days/nights. UI and FI are mostly primary in nature and occur independent of participants’ age and level of adaptive functioning. Solid stool, lower urinary tract symptoms and UTI’s are also common among females with RS. These findings suggest detailed (objective) assessment of incontinence as part of the care for females with RS and which may reveal indications for treatment of incontinence in RS. Until now, no (controlled) studies on the effects of treatment of incontinence in individuals with RS have been published. Since incontinence in females with RS did not differ from incontinence in females in the control group, it may be concluded that incontinence is not part of the behavioral phenotype of RS, but that there is an increased risk for solid stool in females with RS.
